# Current Practice Patterns and Barriers to Intravascular Imaging–Guided Percutaneous Interventions: Insights from a Canadian Nationwide Survey

**DOI:** 10.1016/j.cjco.2026.02.019

**Published:** 2026-02-28

**Authors:** Mehdi Madanchi, Natalia Pinilla-Echeverri, Kevin R. Bainey, Andrea Lavoie, Samer Mansour, Tej Sheth, Matthew Sibbald

**Affiliations:** a Population Health Research Institute, institution, location; b McMaster University and Hamilton Health Sciences, Hamilton, Ontario, Canada; c Mazankowski Alberta Heart Institute, University of Alberta, Edmonton, Alberta, Canada; d Cardiology Division, Department of Medicine, University of Saskatchewan, Regina, Saskatchewan, Canada; e Division of Cardiology, Centre Hospitalier de l’Université de Montréal, Montréal, Québec, Canada

**Keywords:** intravascular imaging, percutaneous coronary intervention, coronary artery disease

## Abstract

**Background:**

Despite robust evidence supporting use of intravascular imaging (IVI) to guide complex percutaneous coronary intervention (PCI), its adoption across Canada remains poorly defined.

**Methods:**

A standardized cross-sectional electronic survey was distributed to catheterization laboratory directors across Canada.

**Results:**

Sites were evenly distributed between academic (9; 43%) and nonacademic (12; 57%) institutions, with a mean annual PCI volume of 1706 ± 722 procedures. IVI integration into the catheterization laboratory was reported in 38 of 56 laboratories (68%), with a median of 6 PCI operators (interquartile range, 5-9) per site. Operator comfort levels were generally good, with the majority of catheterization laboratory directors describing their operators as being somewhat experienced (6; 29%) or very experienced (9; 43%). Most directors (90%) perceived IVI as having a positive impact on outcomes. Overall, results showed a trend toward an increase in IVI-guided PCI between 2022 and 2024 (15.6% vs 19.1%, *P* = 0.7). The most-cited barriers to broader IVI use were increased procedure time (76%), cost of equipment or devices (67%), and reimbursement challenges (48%), with no differences between Ontario and non-Ontario sites (40% vs 33%, *P* = 1.0). Over half (52%) of centres were planning to complete IVI upgrades within the next 2 years.

**Conclusions:**

Although IVI is widely available in Canadian catheterization laboratories, it remains underused, due to barriers related to procedure time, workflow, and cost. A coordinated effort among key stakeholders is needed to reduce current barriers and promote broader guideline-concordant use and IVI adoption.

## Résumé

**Contexte:**

Malgré des preuves solides en faveur de l'imagerie intravasculaire (IIV) pour guider les interventions coronariennes percutanées (ICP) complexes, son adoption à travers le Canada reste mal définie.

**Méthodologie:**

Un questionnaire électronique transversal standardisé a été distribué aux directeurs de laboratoires de cathétérisme à travers le Canada.

**Résultats:**

Les centres étaient répartis de manière équilibrée entre les établissements universitaires (9, soit 43 %) et non universitaires (12, soit 57 %), avec un volume annuel moyen d'ICP de 1 706 ± 722 procédures. L'intégration de l'IIV au sein du laboratoire de cathétérisme a été signalée dans 38/56 (68 %) des laboratoires, avec une médiane de 6 opérateurs d'ICP (intervalle interquartile : 5-9) par site. Le niveau d'aisance des opérateurs était généralement bon, la majorité des directeurs de laboratoires de cathétérisme décrivant leurs opérateurs comme étant plutôt expérimentés (6, soit 29 %) ou très expérimentés (9, soit 43 %). La plupart des directeurs (90 %) estimaient que l'IIV avait un impact positif sur les résultats cliniques. Dans l'ensemble, on a observé une tendance à l'augmentation des ICP guidées par IIV entre 2022 et 2024 (15,6 % contre 19,1 %, *p* = 0,7). Les obstacles les plus souvent cités à une utilisation plus large de l'IIV étaient l'allongement de la durée de l'intervention (76 %), le coût des équipements ou des dispositifs (67 %) et les difficultés de remboursement (48 %), sans différence entre les sites ontariens et non ontariens (40 % contre 33 %, *p* = 1,0). Plus de la moitié (52 %) des centres prévoyaient une mise à niveau de leur IIV au cours des deux prochaines années.

**Conclusions:**

Bien que l'IIV soit largement disponible dans les laboratoires de cathétérisme canadiens, elle reste sous-utilisée en raison d'obstacles liés à l'intervention de la procédure, au flux de travail et au coût. Un effort coordonné entre les principales parties prenantes est nécessaire pour réduire les obstacles actuels et promouvoir une utilisation plus large de l'IIV, conforme aux lignes directrices, favorisant son adoption.

Intravascular imaging (IVI)–guided percutaneous coronary intervention (PCI) for complex coronary lesions has been shown to improve patient outcomes, including reductions in death and myocardial infarction, compared with angiography-guided PCI.^1^ The 2024 European Society of Cardiology (ESC) guidelines upgraded IVI-guided PCI to a class IA recommendation in complex coronary lesions,^2^ whereas the most recent 2025 American College of Cardiology/American Heart Association/Society for Cardiovascular Angiography and Interventions (ACC/AHA/SCAI) guidelines provide a class IIA recommendation for IVI use in acute coronary syndromes.^3^

Despite this strong evidence, real-world utilization of IVI remains highly variable across countries, centres, and operators.^1^^,^^4^ Factors contributing to this heterogeneity include disparities in infrastructure availability, integration of IVI systems within catheterization laboratories, and workflow adaptability.^5^ Additionally, economic and logistical barriers, limited reimbursement frameworks, and increased procedure time have been cited consistently as deterrents to routine use. Training and operator familiarity further compound these challenges, particularly in lower-volume or non-academic centres where opportunities for hands-on experience may be fewer.^6^^,^^7^

Understanding these barriers in the Canadian practice context is crucial for guiding strategies that promote broader and more consistent IVI adoption, as differences in provincial funding models and local availability of hardware and software may contribute to regional heterogeneity. In addition, workflow organization, scheduling pressures, and team dynamics may influence procedural efficiency and operator perceptions.^8^ Given these multifactorial challenges, understanding IVI practice patterns in the Canadian context is essential.

To address these questions, we conducted a national cross-sectional survey of catheterization laboratories directors across Canada to provide an overview of IVI availability, utilization patterns, and integration into daily workflow, while also identifying the principal barriers to its adoption.

## Methods

### Study design, participants, and ethics

We conducted a cross-sectional survey of Canadian catheterization-laboratory directors between January and March 2025 to characterize IVI utilization patterns, barriers, and workflow factors across provinces and laboratory types. Invitations were distributed electronically via professional networks, with a 3-week response window and weekly reminders. All catheterization laboratory directors from academic and nonacademic centres were invited to take part in the survey. The study protocol received formal approval from the Hamilton Integrated Research Ethics Board (HiREB) under Protocol #18326.

### Survey structure and main variables

A structured questionnaire was developed by expert consultation, covering the following areas: (i) IVI utilization patterns; (ii) perceived barriers (cost, time, reimbursement, training, perceived benefit); and (iii) workflow affordances.

### Data analysis

We used descriptive statistics (mean with standard deviation, or median with interquartile range for continuous variables, counts with percentages for categorical variables) and exploratory comparative tests (*t*-test or Wilcoxon/Kruskal–Wallis test for continuous data; χ^2^ or Fisher’s exact test for categorical data), with a 2-sided α = 0.05. Qualitative free-text responses underwent thematic analysis. Predictors of IVI-guided PCI use were assessed using multiple linear regression. All analyses were performed with R (R Foundation for Statistical Computing, Vienna, Austria).

## Results

Of the 47 centres invited to participate, responses were received from 21 centres (45%), representing Ontario (n = 10; 47.6%), Quebec (n = 6; 28.6%), and Alberta (n = 2; 9.5%), and one site each from Manitoba, British Columbia, and New Brunswick (4.8%). Nearly half of the catheterization laboratory directors (9; 43%) who participated in the survey had 0-5 years of experience in this position. The participating centres were a mix of 9 (43%) from academic sites and 12 (57%) from nonacademic sites. The mean annual PCI volume among all participating centres was 1706 ± 722 cases. Sites reported a median of 6 PCI operators (IQR 5-9) and 2 laboratories per site (IQR 2-3), with a total of 56 laboratories. Among the participating centres, 19 catheterization laboratories (90%) reported having intravascular ultrasound (IVUS), and 18 (86%) reported having optical coherence tomography capabilities, respectively. Among IVUS users, most centres had non–high definition IVUS (13of 19; 68%), and among OCT users, the majority used the Ultreon 2.0 system (Abbott, Abbott, IL) (13/18, 86%). Integration of the IVI system into the catheterization laboratory was reported in 38 (68%) of the laboratories. Further characteristics about the participating centres and the type of IVI modalities can be found in Table 1.

**Table 1:** Baseline characteristics of participating catheterization laboratories

Variable	Value
**Years as catheterization lab director**	
0–5	9 (42.9)
6–10	9 (42.9)
11–15	1 (4.8)
16–20	1 (4.8)
> 20	1 (4.8)
**Province**	
Ontario	10 (48)
Quebec	6 (20)
Alberta	2 (9.5)
Manitoba	1 (4.8)
British Columbia	1 (4.8)
New Brunswick	1 (4.8)
**Type of catheterization laboratory**	
Academic site	9 (43)
Nonacademic site	12 (57)
**Annual PCI volume**	1706 ± 722
**PCI operators per laboratory**	7.0 ± 3.2
**Number of laboratories per site**	2.7 ± 1.1
**IVUS availability**	16 of 19 (84)
Philips (Philips, Amsterdam, the Netherlands)	11 of 16
Boston Scientific (Boston Scientific, MA)	8 of 16
Bracco ACIST HDi (Bracco Imaging, MN)	5 of 16
Volcano (Philips, San Diego, CA)	3 of 16
Polaris (Boston Scientific, MA)	1 of 16
**OCT availability**	18 of 21 (86)
Ultreon 2.0	13 of 18
Ultreon 1.0	5 of 18
**IVI integration**	38 of 56 (68)

Data are mean (± standard deviation), or number (percentage), as appropriate.

IVI, intravascular imaging; IVUS, intravascular ultrasound; OCT, optical coherence tomography; PCI, percutaneous coronary intervention.

**Figure 1:**
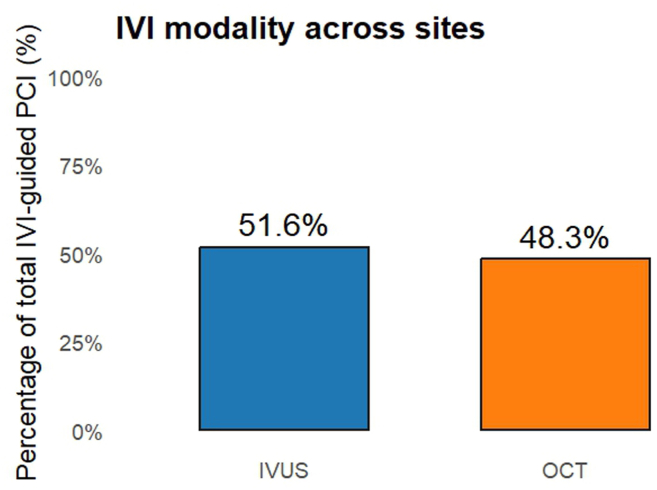
Intravascular imaging (IVI) modalities across sites. IVUS, intravascular ultrasound; OCT, optical coherence tomography; PCI, percutaneous coronary intervention.

**Figure 2:**
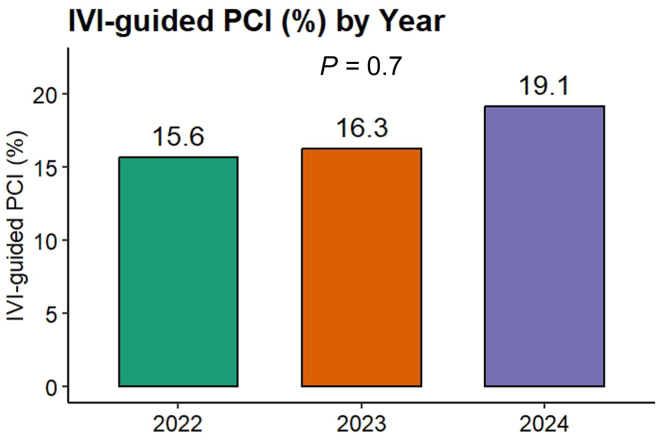
Intravascular imaging (IVI) volume by year. PCI, percutaneous coronary intervention.

The distribution of IVI modalities across centres was substantial and nearly equal, with IVUS and OCT accounting for 51.6% and 48.3% of use, respectively (*P* = 0.85), as reported in Figure 1. Additionally, a modest trend occurred toward increased IVI-guided PCI between 2022 and 2024 (15.6% vs 19.1%, *P* = 0.7), as reported in Figure 2*,* and this pattern remained consistent in a sensitivity analysis restricted to Ontario centres (Supplemental Fig. S1). IVI utilization was not significantly associated with academic status, number of operators, PCI volume, site type, or region (overall model *P* = 0.21; Supplemental Table S1). We also tested the association between IVI utilization in 2024 and years of experience as a catheterization laboratory director. Considering the small number of responses, the analysis showed a trend toward a non–statistically significant association—an increase in years of experience appeared to be associated with a decrease in IVI utilization in 2024. However, this relationship was not statistically significant (*P* = 0.43), as illustrated in Supplemental Table S2 and Supplemental Figure S2.

The most-frequent indications for IVI-guided PCI included left main (100%), calcified lesions (90%), in-stent restenosis (81%), chronic total occlusion (57%), and bifurcation (38%) PCIs. Most directors reported that their operators were somewhat (43%) or very experienced (29%) with IVI. The perceived effect on procedure duration was substantial, with 71% of the directors indicating a moderate or major impact. Most of the respondents viewed IVI as beneficial to patient outcomes, with 38% rating the impact as strongly positive, and 52% rating it as somewhat positive. More than half of participating centres had a plan to upgrade or expand IVI capability within the next 2 years. Further details about IVI utilization patterns, operator experience, and perceived impact on outcomes can be found in Table 2.

**Table 2:** Intravascular imaging utilization patterns, operator experience, and perceived impact on outcomes

Variables	Value
**Indications for IVI use**	
Left main	21 of 21 (100)
Calcified lesions	19 of 21 (90)
ISR	17 of21 (81)
CTO	12 of 21 (57)
Bifurcations	8 of 21 (38)
ACS	6 of 21 (29)
**Operator comfort with IVI use**	
Very experienced	6 (29)
Somewhat experienced	9 (43)
Neutral	4 (19)
Somewhat inexperienced	2 (9.5)
**Impact of procedure time**	
No impact/minor	6 (29)
Moderate	8 (38)
Major	7 (33)
**Perceived impact on outcomes**	
Strongly positive	8 (38)
Somewhat positive	11 (52)
Neutral	2 (10)
**Plans to upgrade in the 2-Y horizon**	
Yes	11 (52)
Possibly	6 (29)
No	4 (19)

Data are number (percentage).

ACS, acute coronary syndrome; CTO, chronic total occlusion; ISR, in-stent restenosis; IVI, intravascular imaging.

**Figure 3:**
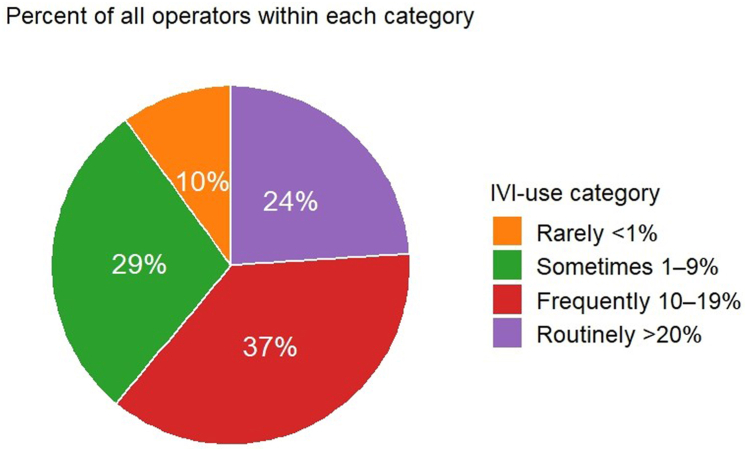
Frequency of intravascular imaging use across operators.

**Figure 4:**
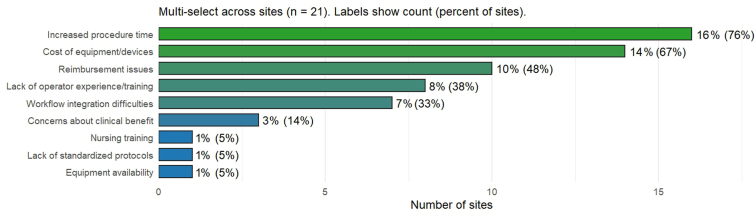
Primary barriers to intravascular imaging use.

Patterns of IVI use varied across operators, with the pooled distribution skewed toward frequent or routine use, as depicted in Figure 3. The most-cited barriers to routine IVI use were operational and economic. Increased procedure time (76%) and equipment and/or device cost (66%) were cited most frequently, followed by reimbursement challenges (48%), operator training and/or experience (38%), and workflow integration issues (33%); less common were concerns about clinical benefit (14%), nursing training needs (5%), equipment availability (5%), and lack of standardized protocols (5%), as reported in Figure 4. Among the respondents, 67% highlighted the need for additional training programs, continuing medical education opportunities, and industry involvement as key factors to increase the use and integration of IVI into daily practice.

## Discussion

This national survey provides an overview of the current state of IVI use in Canadian catheterization laboratories. Although IVI capability is broadly available, its integration into routine practice remains inconsistent, reflecting a combination of economic, operational, and educational challenges. Overall, this study highlights 3 key themes: (i) limited adoption of IVI despite its broad availability; (ii) persistent operational and financial barriers; and (iii) a growing institutional commitment to expansion supported by operator confidence and perceived clinical value.

Despite the vast majority of centres having access to IVI, and more than two-thirds of laboratories reporting having integrated IVI systems, routine adoption remains limited, with IVI used in about 20% of all PCI procedures in 2024. Compared with other regions, the level of IVI use in Canada is similar to that in the US and many European countries, yet it remains far behind that in countries such as Japan and South Korea, where IVI use exceeds 90%.^9^ Although the variation seen in this survey may in part reflect differences in case mix and the proportion of complex lesions across centres, it more likely stems from differences in institutional culture and operator experience. In addition, time pressures and cost considerations may discourage broader application of IVI, even in settings with well-integrated imaging systems.

The most frequently reported obstacles to broader IVI implementation were increased procedure time (76%), device cost (66%), and reimbursement limitations (48%), aligning with trends described in the contemporary literature.^9^^,^^10^ In addition to being identified as a key barrier to broader IVI adoption, the perceived impact of IVI use on procedural duration was substantial among catheterization laboratory directors. Integrated imaging systems and standardized workflows, such as IVUS 1-2-3 and Media–Lumen–Diameter, Morphology, Length, and Diameter–Matching, Apposition, and eXpansion (MLD MAX) algorithms,^11^ represent practical solutions to mitigate the time increases associated with IVI. These structured approaches may facilitate image acquisition, interpretation, and decision-making, allowing IVI to be incorporated efficiently within routine PCI workflows.

Economic barriers, by contrast, are more structural and are especially relevant within Canada’s publicly funded healthcare system. The absence of dedicated billing codes or bundled reimbursement in many provinces likely discourages routine IVI use. However, health-economic analyses from Australia and the United Kingdom, which have a reimbursement system similar to the one in Canada, have shown that IVI is cost-effective over time.^12^^,^^13^ Introduction of supplemental reimbursement for IVI-guided PCI, as recently implemented in the province of Ontario, may be an example on how to reduce financial disincentives and promote more uniform adoption across centres. Additionally, addressing these structural barriers through national funding frameworks or by incorporating IVI into quality-based incentive programs could substantially enhance guideline-concordant use across Canada. Finally, the IVI reimbursement pathway, whether directed to the hospital or the physician, and its impact on IVI adoption, remain speculative. Hospital-based reimbursement may allow for more structured and potentially cost-effective implementation, but it also can be slower to adapt, due to bureaucratic processes, which may hinder the adoption of newer technologies such as IVI. In contrast, physician reimbursement, particularly in fee-for-service systems, is more closely aligned with the added workload and may serve as a more immediate incentive, potentially driving faster adoption. However, this model also presents the risk of overuse or inappropriate use if it is not carefully regulated. Additionally, political complexities further complicate the situation, influencing the practical application of these reimbursement structures.

Operators’ level of confidence in and perceived benefit of IVI were high, as nearly 90% of operators considered IVI beneficial or strongly beneficial to patient outcomes. More than half of participating centres reported having plans to expand or upgrade their IVI capabilities within the next 2 years, reflecting substantial institutional investment aligned with the growing evidence base and contemporary guideline recommendations.^2^^,^^3^ Together with strong operator engagement, this institutional commitment may provide a solid foundation for broader IVI adoption as workflow efficiency and training continue to improve. Canada’s publicly funded model may uniquely amplify economic barriers, given that system-level decision-making is often less responsive to the integration of upfront technologies, even when such investments have clear potential to reduce downstream costs and improve outcomes. Although IVI-guided PCI increased between 2022 and 2024, likely influenced by emerging evidence and updated guidelines,^1^, ^2^, ^3^^,^^14^, ^15^, ^16^ considerable work remains to close the gap between IVI availability and evidence-based use. This need highlights the fact that investing in equipment alone is not enough to ensure guideline-concordant IVI-guided PCI use. Based on recently published data from a large sample at a tertiary Canadian PCI centre, where complex PCI accounted for approximately 50% of all procedures, the goal for the IVI-guided PCI rate across all Canadian PCI centres should be between 25% and 50%.^17^ This target range acknowledges that IVI delivery may not always be feasible, due to factors such as equipment deliverability, vessel tortuosity, and other procedural challenges.

Beyond political and industry stakeholders, professional organizations such as the Canadian Association of Interventional Cardiology (CAIC) will be key to systematically assessing the barriers to IVI use, developing practical solutions, and promoting evidence-based adoption among physicians. In fact, more than two thirds of the respondents identified the need for additional training programs, continuing medical education, and industry involvement as essential components for increasing IVI adoption. These educational sessions were seen as crucial for improving operator experience and comfort with IVI technologies, ultimately supporting their integration into daily practice. Such coordinated leadership is essential to translating the current availability of IVI into consistent, high-quality practice.

Overall, our findings depict a healthcare environment with technical capacity and professional motivation but also economic and structural constraints. Although IVI capability is broad, routinization of use remains incomplete. National strategies should prioritize the following 3 key actions: (i) aligning reimbursement policies to remove economic disincentives; (ii) expanding operator education; and (iii) enhancing workflow integration and artificial intelligence–based image analysis. Additionally, industry stakeholders play an important role in facilitating broader adoption by ensuring fair pricing, which could help reduce costs and improve accessibility across centres. These measures could collectively reduce variability, streamline procedures, and improve patient outcomes by embedding IVI into routine PCI decision-making.

### Limitations

This study has several limitations. First, responses were obtained from 21 of 47 centres (45%), meaning less than half of the eligible centres participated in the survey. Additionally, some medium to large PCI centres did not participate. As a result, the findings cannot be generalized and must be interpreted with caution, as they may not fully represent the diversity of practices across all Canadian PCI centres.

Although the study focused on catheterization laboratory director experience, individual operator-level experience and training were not captured directly, which may limit the ability to draw inferences regarding operator-specific factors influencing IVI utilization. In addition, the data were self-reported and may be subject to recall or response bias. Finally, the descriptive and survey-based nature of the study captures practices at a single time point, meaning causal or temporal inferences beyond the reported period cannot be made. As a result, observed associations should be viewed as reflective rather than explanatory.

## Conclusions

IVI is widely available across Canadian catheterization laboratories, and its clinical value is well recognized. Nevertheless, IVI remains underused, with persistent variability in adoption driven largely by barriers related to procedure time, operator workflow, and cost. Investment in workflow optimization, supportive reimbursement policies, and training pathways for all levels is key to promoting broader IVI adoption nationwide and equitable access for all patients.

## Ethics Statement

The research reported in this paper adhered to the CROSS guidelines.

## Patient Consent

The authors confirm that patient consent was not required for this study. The study protocol received formal approval from the Hamilton Integrated Research Ethics Board (HiREB) under Protocol #18326.

## Funding Sources

The authors have no funding sources to declare.

## Disclosures

M.M. reports receiving a research grant from the Swiss National Science Foundation (SNSF). N.P.-E. reports serving as a consultant and speaker, receiving research grant support, and serving on the Advisory Board for Abbott Vascular. The other authors have no conflicts of interest to disclose.

## References

[1] Stone G.W., Christiansen E.H., Ali Z.A. (2024). Intravascular imaging-guided coronary drug-eluting stent implantation: an updated network meta-analysis. Lancet.

[2] Vrints C., Andreotti F., Koskinas K.C. (2024). 2024 ESC guidelines for the management of chronic coronary syndromes. Eur Heart J.

[3] Rao SV, O’Donoghue ML, Ruel M (2025). ACC/AHA/ACEP/NAEMSP/SCAI guideline for the management of patients with acute coronary syndromes: a report of the American College of Cardiology/American Heart Association Joint Committee on Clinical Practice Guidelines. J Am Coll Cardiol.

[4] Sibbald M, Cioffi GM, Shenouda M (2024). Intravascular imaging in the diagnosis and management of patients with suspected intracoronary pathologies: a CJC white paper. Can J Cardiol.

[5] Bergmark B., Dallan L.A.P., Pereira G.T.R. (2022). Decision-making during percutaneous coronary intervention guided by optical coherence tomography: insights from the LightLab Initiative. Circ Cardiovasc Interv.

[6] Raja A., Osborn E.A., Bergmark B.A. (2022). OCT utilization: summary statistics from the LightLab clinical initiative. Catheter Cardiovasc Interv.

[7] Khuddus M.A., Darki A., Padaliya B.B. (2022). Improving efficiency and operator proficiency during percutaneous coronary interventions utilizing a standardized optical coherence tomography workflow. Catheter Cardiovasc Interv.

[8] Stein E.J., Mesenbring E., Smith T. (2025). Intravascular imaging as a performance measure for percutaneous coronary intervention. Circ Cardiovasc Interv.

[9] Koskinas K.C., Nakamura M., Räber L. (2018). Current use of intracoronary imaging in interventional practice—results of a European Association of Percutaneous Cardiovascular Interventions (EAPCI) and Japanese Association of Cardiovascular Interventions and Therapeutics (CVIT) clinical practice survey. EuroIntervention.

[10] Mintz G.S. (2024). Intravascular imaging for PCI: Do protocols matter?. JACC Cardiovasc Interv.

[11] Ali Z.A., Karimi Galougahi K., Mintz G.S. (2021). Intracoronary optical coherence tomography: state of the art and future directions. EuroIntervention.

[12] Zhou J., Liew D., Duffy S.J. (2021). Intravascular ultrasound versus angiography-guided drug-eluting stent implantation: a health economic analysis. Circ Cardiovasc Qual Outcomes.

[13] Sharp A.S.P., Kinnaird T., Curzen N. (2024). Cost-effectiveness of intravascular ultrasound-guided percutaneous intervention in patients with acute coronary syndromes: a UK perspective. Eur Heart J Qual Care Clin Outcomes.

[14] Lee J.M., Choi K.H., Song Y.B. (2023). Intravascular imaging-guided or angiography-guided complex PCI. N Engl J Med.

[15] Holm N.R., Andreasen L.N., Neghabat O. (2023). OCT or angiography guidance for PCI in complex bifurcation lesions. N Engl J Med.

[16] Ali Z.A., Landmesser U., Maehara A. (2023). Optical coherence tomography-guided versus angiography-guided PCI. N Engl J Med.

[17] Madanchi M, Pinilla-Echeverri N, Mehta SR (2025). Use of intravascular imaging to guide percutaneous coronary interventions: experience from a single, high-volume Canadian centre. CJC Open.

