# Supplementary material for: Current Practice Patterns and Barriers to Intravascular Imaging–Guided Percutaneous Interventions: Insights from a Canadian Nationwide Survey
Source: CJC Open. 2026 Feb 28;8(6):729–35. doi: 10.1016/j.cjco.2026.02.019 (PMC13282516; doi:10.1016/j.cjco.2026.02.019)

| **Predictor** | **Estimate** | **95% CI** | **p-value** |
| --- | --- | --- | --- |
| Intercept | 11.48 | −11.4-34.4 | 0.27 |
| PCI volume | −0.0026 | −0.0129-0.0077 | 0.57 |
| Number of operators | 1.47 | −0.64-3.58 | 0.14 |
| Academic site | 7.63 | −8.17-23.4 | 0.29 |
| Region (Quebec) | 14.8 | −35.9- 6.25 | 0.14 |
| Other regions (Ontario, Manitoba, BC) | NA | NA | NA |

**Supplemental Table S1**. Predictors of Intravscular Imaging-Guided Percutaneous Coronary Intervention Use Across Canadian Catheterization Laboratories

**Supplemental Table S2.** Regression Analysis of Intravascular Imaging Use Percentage by Years of Experience Across Canadian Catheterization Laboratories

| **Variable** | **Estimate** | **Standard Error** | **p-value** |
| --- | --- | --- | --- |
| **Years in Practice (0-5 years)** | 26.977 | 5.152 | 0.00195 |
| **Years in Practice (6-10 years)** | -7.511 | 7.286 | 0.34234 |
| **Years in Practice (More than 20 years)** | -13.910 | 11.520 | 0.27269 |

**Supplemental Figure S1**. Predictors of IVI-Guided PCI by year in Ontario


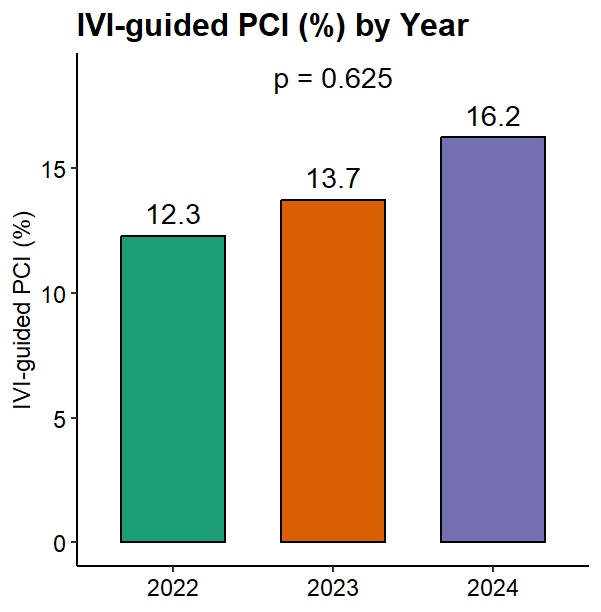


**Supplemental Figure S2**. Intravascular Imaging-Guided PCI Use by Years of Experience of the Cath Lab Director in 2024


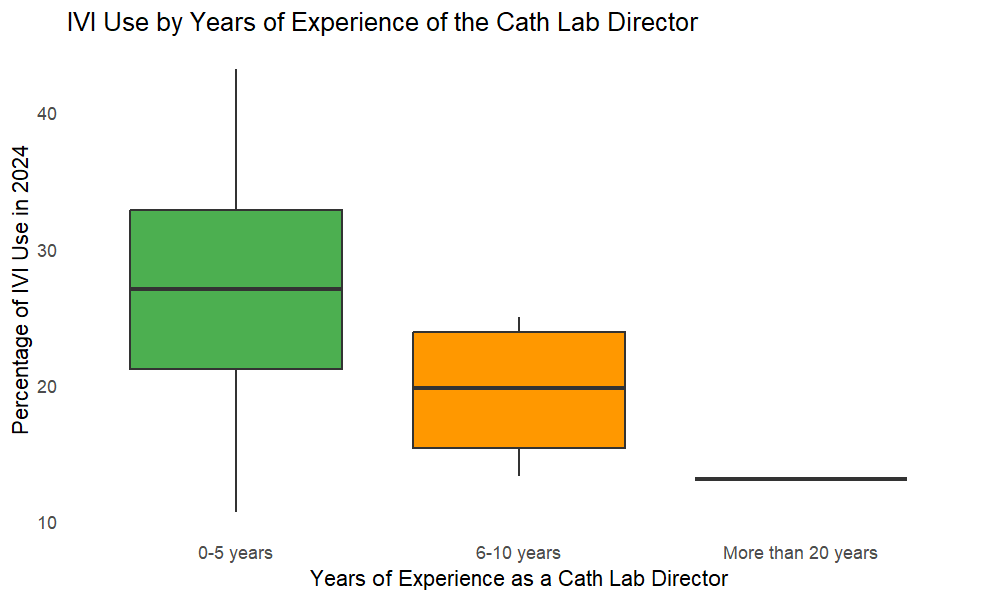

Supplement: Supplementary Table [file mmc1.docx]
